# Respiratory Tract: Affections, Symptoms and Treatment

**Published:** 1904-11

**Authors:** Arthur B. Smith

**Affiliations:** Springfield, O.


### RESPIRATORY TRACT: AFFECTIONS, SYMPTOMS AND TREATMENT.

BY DR. ARTHUR B. SMITH, SPRINGFIELD, O.

The average physician is frequently vexed in finding a condition which resists his best efforts to bring about a cure. This holds good in almost every disease at some time or other, but particularly in affections of the respiratory tract, where there may be a great variety of symptoms in several cases of the same disease.

Almost every physician has some favorite prescription for coughs, bronchitis, laryngitis, etc., which he uses until suddenly it seems to lose its efficacy—why, no one knows. Then another remedy takes its place until it, too, fails to give the desired result. It is rarely that one finds a cough remedy which will be consistently good in the majority of cases. Theoretically there appears to be a well-founded objection to the use of cough-syrups in general, but nevertheless there are times when nothing else gives satisfaction; therefore, the physician pins his faith to that remedy from which he and his patients derive the most good. It is not always easy to find such a remedy, but when it is once found, it is equally difficult to dispense with, and often the physician is almost compelled to resort to a routine treatment. In such cases, of course, he wants the best.

There are constantly being placed on the market new formulas for affections of the air-passages. Some of these formulas are of undoubted benefit in some cases, but usually it will be found that the results are far from satisfactory. Many of them can not be taken when there is any gastric complication, as is sometimes the case, because of consequent nausea and vomiting. Others seem almost invariably to act as cardiac depressants and are highly objectionable for that reason. With the advent of heroin, however, these disagreeable features have, to a great extent, been avoided. Heroin, in the vast majority of cases, can be tolerated by even the most sensitive stomach, and, if any disturbance should occur, it can easily be obviated by decreasing the dosage and then gradually resuming the previous amount. Heroin can be prescribed, in cases which are complicated by an enfeebled heart, without danger of depressing effects. As compared with codeine, its sedative action on the respiration is much more powerful. The fatal dose of heroin is said to be one hundred times the efficacious dose, while with codeine the efficacious dose is one tenth of the fatal dose. In other words, heroin is ten times safer than codeine, and can be given in such larger doses, if necessary, without danger. It appears to exert a specific action on the center of respiration without causing disturbances of any other organs or centers, and there is no danger of acquiring any habit by its use.

In phthisical patients the well-known lack of appetite and intolerance of various foods render it imperative to give remedies which will not in any way interfere with the digestive functions, while at the same time controlling or alleviating the cough and other distressing conditions.

Some time ago my attention was called to a preparation composed of a solution of heroin in glycerine, combined with expectorants, called Glyco-Heroin (Smith). Each teaspoonful of this preparation contains one-sixteenth grain of heroin by accurate dosage. It is of agreeable flavor, therefore easy to administer to children, for whom the dose can be easily reduced with any liquid, or by actual measurement. It possesses many advantages not shown by any other preparation I have used, and has none of their disagreeable features.

In citing some of the cases treated with this remedy I shall not go into a minute description of any case, but briefly state the conditions which existed and the results obtained, which were uniformly good.

Case 1. S. B., aged 16. Caught a severe cold while traveling. This developed into an unusually severe attack of bronchitis with mucous rales, pain, cough and some slight fever. Prescribed Glyco-Heroin (Smith) one teaspoonful every two hours, decreased to every three hours. After a few doses were taken there was a decided improvement, the respirations were slower and deeper, the expectoration freer and the temperature normal. In a few days the patient was practically well and able to return to school. No medicine except Glyco-Heroin (Smith) was given and the results from its use were excellent.

Case 2. W. L., aged 31. Acute bronchitis. Painful cough, with difficult expectoration, particularly when in a reclining posture. Glyco-Heroin (Smith) in teaspoonful doses every three hours gave speedy relief and a cure was effected in a few days.

Case 3. S. W., age 60. Chronic bronchitis. He coughed for years, with expectorations of a thick, yellow purulent and very offensive matter. Had lost flesh gradually until about twenty pounds below usual weight. No appetite, very constipated, pains all over chest, night sweats and insomnia. Patient on the verge of nervous prostration and greatly weakened. She was given bromides, a tonic, and Glyco-Heroin (Smith), the latter in the usual dose at intervals of two hours. The first few doses were not well borne, as they seemed to cause some nausea, but by giving a smaller dose and then gradually increasing it, tolerance was soon obtained, and the results were remarkable. The cough and expectoration greatly decreased, the appetite improved and the patient became much better in every way. The treatment was continued as before, except that the Glyco-Heroin (Smith) was given every three hours. In three weeks the patient was eating almost everything she pleased and sleeping well. The night sweats had stopped, together with the cough, and, as the patient expressed it, she "felt like another woman." At present she is in perfect health and needs no medicine except an occasional laxative.

Case 4. B. E., aged 26. Severe bronchitis accompanying an attack of influenza. Various remedies were tried in this case, with negative results, until Glyco-Heroin (Smith) was given in teaspoonful doses every three hours. In a short time decided relief was obtained and cough stopped permanently.

Case 5. R. L., aged 6. Capillary bronchitis with pains over chest, cough and difficult expectoration. Glyco-Heroin (Smith) administered fifteen drops every three hours. After taking a few doses the condition was much improved, and a speedy return to perfect health followed.

Case 6. W. H., aged 5. Whooping cough. Spasmodic paroxysms of coughing, sometimes being so severe as to cause vomiting. Tenacious mucus was present, requiring great expulsive effort to loosen it. There was little fever, but the patient was much prostrated and weakened by the cough. Glyco-Heroin (Smith) was given in ten-drop doses every two hours with good results. This was combined with hygienic treatment, the patient being given as much fresh air as possible. In a few days the condition was much ameliorated, the cough under fair control, expectoration was freer and easier to raise, and convalescence uneventful. The case was discharged cured and there were no unpleasant sequelæ, the patient at present being in perfect health.

